# Supplementary material for: Human pancreatic microenvironment promotes β-cell differentiation via non-canonical WNT5A/JNK and BMP signaling
Source: Nat Commun. 2022 Apr 12;13:1952. doi: 10.1038/s41467-022-29646-1 (PMC9005503; doi:10.1038/s41467-022-29646-1)
Supplement: Supplementary file 1 — Supplementary Information [file 41467_2022_29646_MOESM1_ESM.pdf]

Supplemental information:

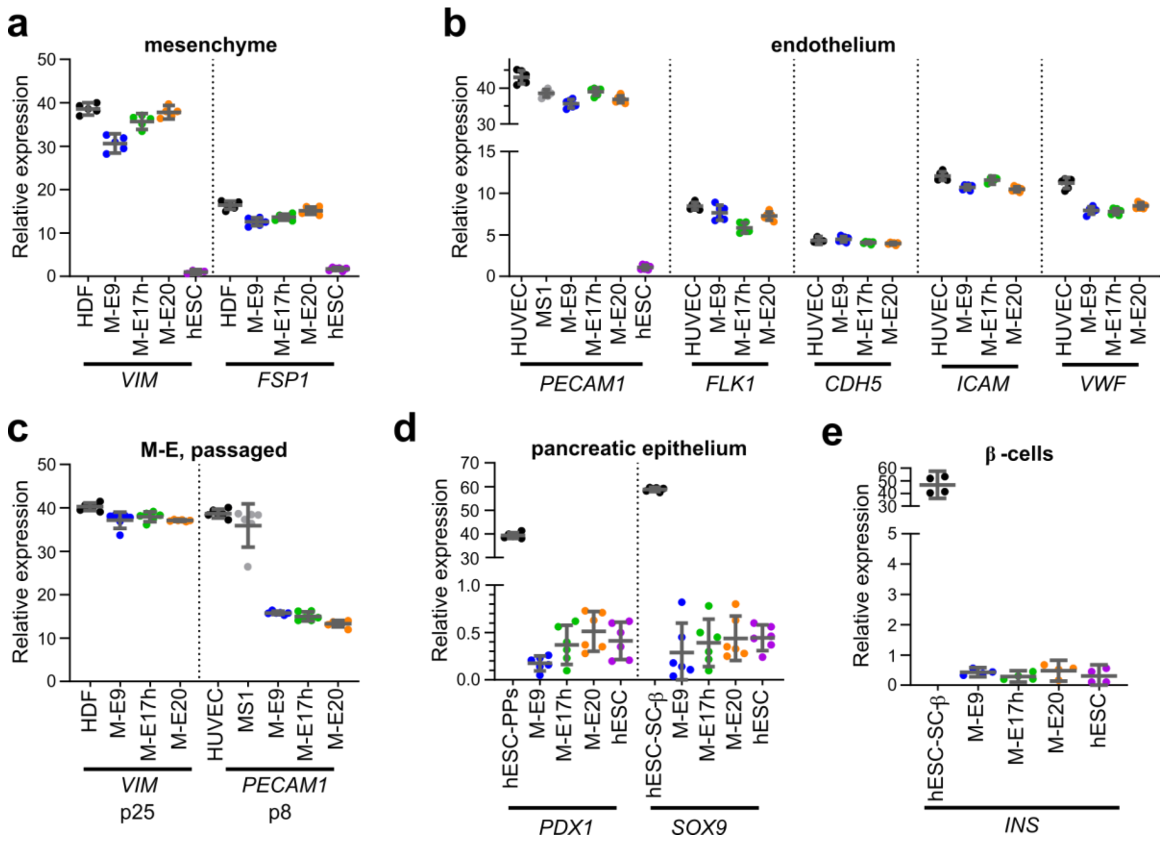

Supplementary Figure 1. Primary cells derived from fetal pancreas at different stages express mesenchymal and endothelial markers but not epithelial markers

a. and b. Characterization of *de novo* derived pancreatic M-E primary cells by qRT-PCR analysis of a. mesenchymal (vimentin, *VIM; FSP1*) and b. endothelial (*PECAM1; FLK1;* VE- cadherin, *CDH5; ICAM; VWF*) markers expression in M-E9, 17.5h and M-E20 cells, and control HUVEC and HDF cell lines, at passage 5. Gene expression was normalized to *TBP*. Lines represent mean ± 95% CI from 6 independent experiments, except for VIM (N = 5, for hESC N = 4), CDH5 HUVEC (N = 4) and ICAM M-E17h (N = 4).

c. *VIM* and *PECAM1* expression analysis by qPCR at passage 25 and passage 8 (respectively) in control mesenchymal cells, HDFs and endothelial cell lines, HUVECs and MS1, along with M-E9, 17.5h and M-E20 cells. Data are presented as mean ± 95% CI from 6 independent experiments.

d. Pancreatic epithelial cell markers, *PDX1* and *SOX9* expression by qRT-PCR in hESC-derived PPs or SC-β cells, M-E9, 17.5h, M-E20 and hESCs. Data are presented as mean ± 95% CI, N = 6 independent experiments.

e. *INS* expression analysis by qPCR in hESC-PPs, M-E9, M-E17.5h, M-E20 and hESCs. Data are presented as mean ± 95% CI, N = 4 independent experiments.

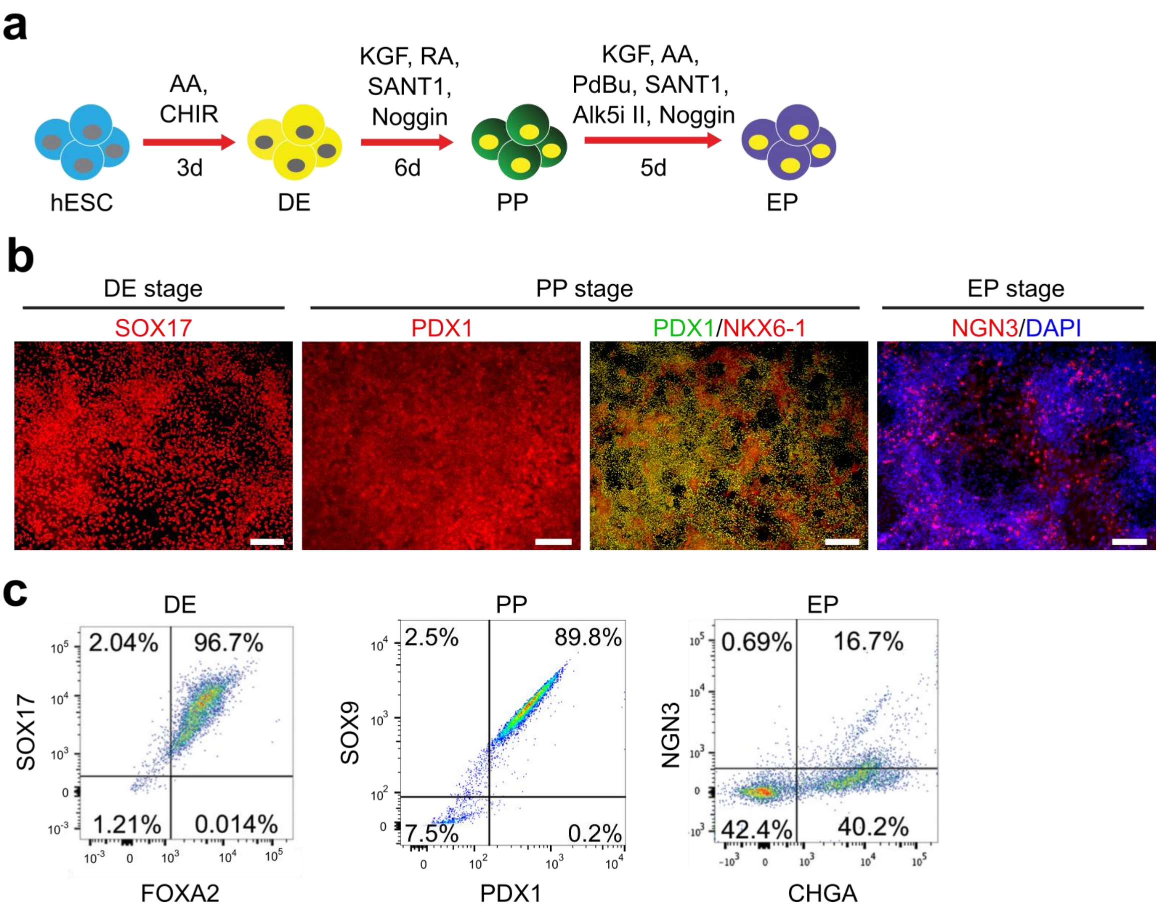

Supplementary Figure 2. Differentiation of hESCs towards pancreatic endocrine progenitors.

a. Overview of the differentiation protocol used to derive pancreatic progenitors (PPs) or endocrine progenitors (EPs) from hESCs.

b. Representative images of definitive endoderm (DE) stained for SOX17, PPs stained for PDX1 (green) and NKX6.1 (red), and EPs stained for NGN3 (red). DAPI marks nuclei in blue. Scale bar = 250 µm. N=12 independent experiments.

c. Flow cytometry analysis and quantification of markers for each stage of the differentiation.

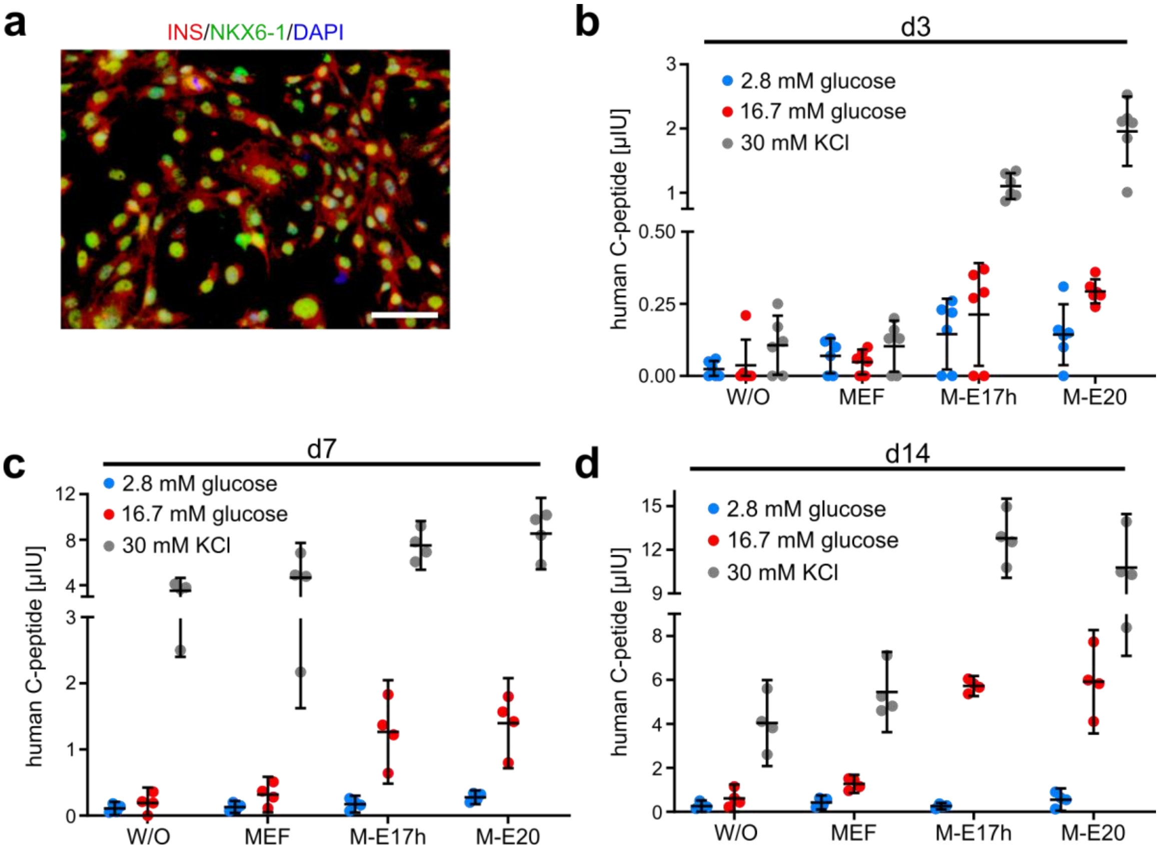

Supplementary Figure 3. Glucose stimulated insulin secretion (GSIS) at d3, 7 and 14 of coculture

a. INS+ (red) cells induced by coculture (d14) with M-E20 cells co-express NKX6.1 (green). Cell nuclei are stained by DAPI (blue). Scale bar = 50 µm. N=6 independent experiments.

b. GSIS results at day 3 of pancreatic progenitor (PP) coculture with M-E cells or MEFs controls. Cells were challenged with 2.8 mM and 16.7 mM glucose. After low/high glucose stimulation, cells were depolarized with 30 mM KCl, and the secreted human C-peptide was measured by ELISA (Mercodia). Results were normalized to cell number and total protein content. Data are represented as mean ± SEM. N = 6 independent experiments and three technical replicates.

c. GSIS results at day 7 of the coculture as described above, except the different range of Y-axis scale. N = 4 independent experiments and three technical replicates.

d. GSIS results at day 14 of the coculture as described above, except the different range of Y-axis scale. N = 4 independent experiments and three technical replicates.

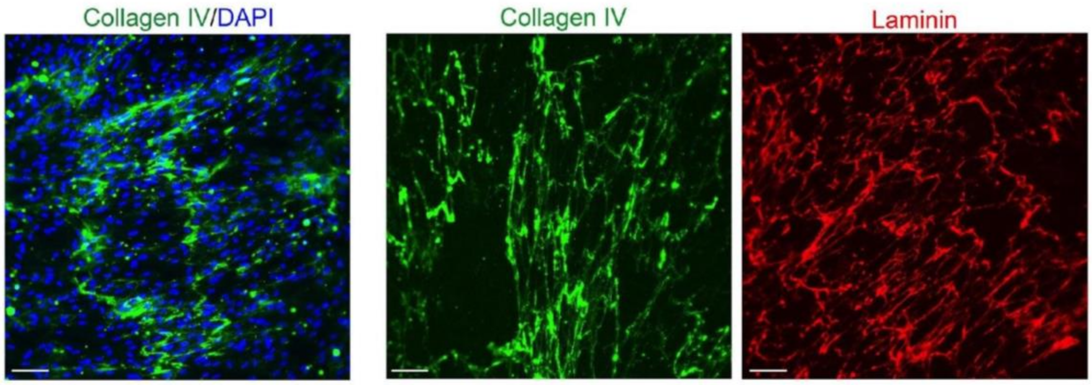

Supplementary Figure 4. Characterization of ECM from M-E20 cells.

(Left panel) Collagen IV (green) is present in ECM of M-E20 cells, the nuclei of which are stained with DAPI (blue). (Middle and right panels) For experiments described in Figures 2B and D M-E20 cells were removed by decellularization, as proved by loss of DAPI-stained nuclei, whereas ECM components Collagen IV (green, middle panel) and Laminin (red, right panel) were preserved in the cell-depleted matrix. Scale bar = 100 μm. N=4 independent experiments.

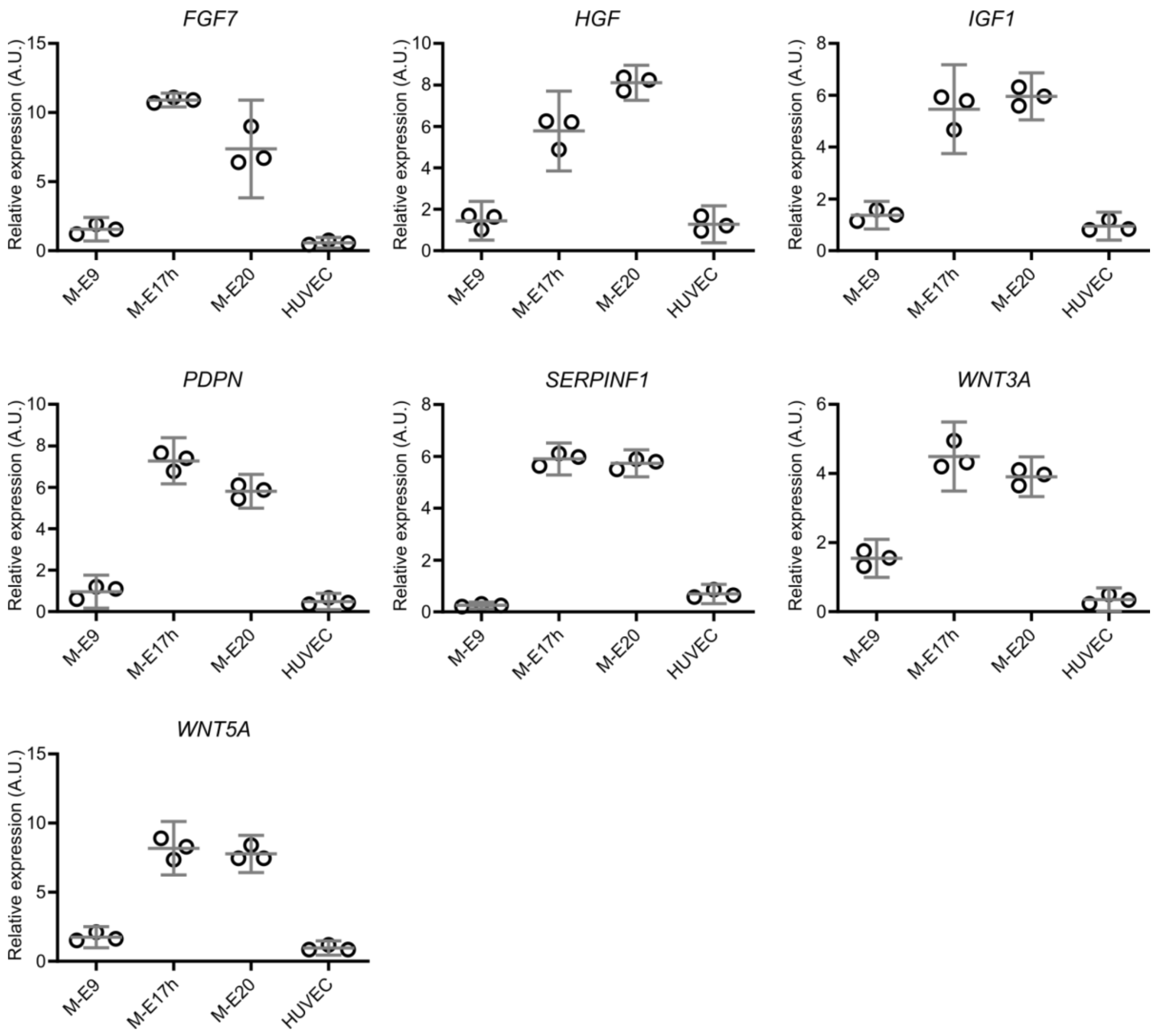

Supplementary Figure 5. Enrichment of the selected factors in M-E17h and M-E20 cells

qRT-PCR analysis of the selected factors in M-E9, M-E17h, M-E20 cells and HUVECs. Lines denote mean ± 95% CI. Data was normalized to TBP. N = 3 independent experiments.

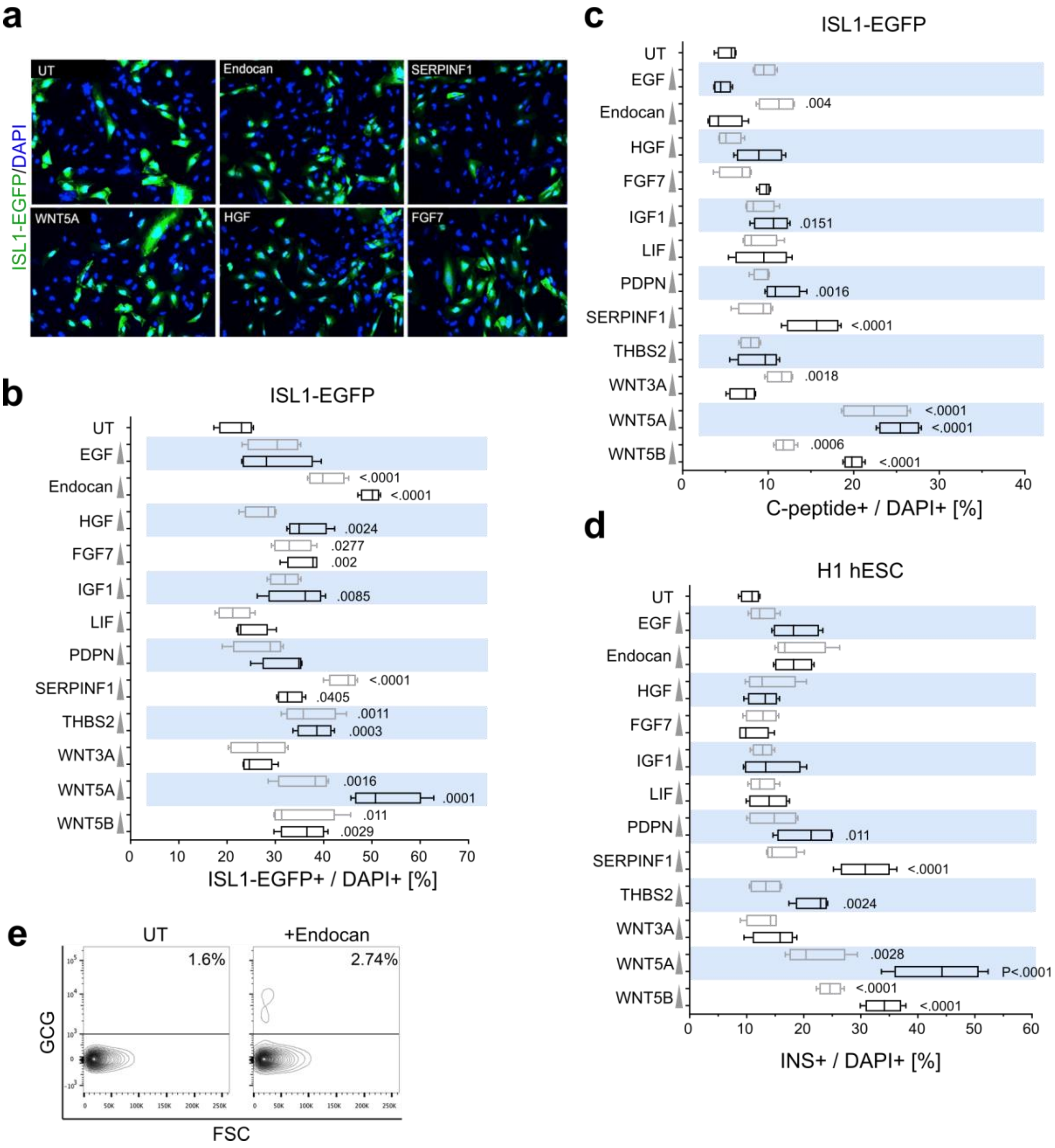
a
UT
Endocan
SERPINF1
WNT5A
HGF
FGF7
ISL1-EGFP/DAPI
b
ISL1-EGFP
UT
EGF
Endocan
HGF
FGF7
IGF1
LIF
PDPN
SERPINF1
THBS2
WNT3A
WNT5A
WNT5B
<.0001
<.0001
.0024
.0277
.002
.0085
<.0001
.0405
.0011
.0003
.0016
.0001
.011
.0029
0 10 20 30 40 50 60 70
ISL1-EGFP+ / DAPI+ [%]
c
ISL1-EGFP
UT
EGF
Endocan
HGF
FGF7
IGF1
LIF
PDPN
SERPINF1
THBS2
WNT3A
WNT5A
WNT5B
.004
.0151
.0016
<.0001
.0018
.0006
<.0001
0 10 20 30 40
C-peptide+ / DAPI+ [%]
d
H1 hESC
UT
EGF
Endocan
HGF
FGF7
IGF1
LIF
PDPN
SERPINF1
THBS2
WNT3A
WNT5A
WNT5B
.011
<.0001
.0024
.0028
P<.0001
<.0001
<.0001
0 10 20 30 40 50 60
INS+ / DAPI+ [%]
e
UT
+Endocan
1.6%
2.74%
GCG
FSC

Supplementary Figure 6. Pancreatic primary M-E cells signals promote β-cell development.

a. Differentiated ISL1-EGFP cells stained with GFP antibody after Endocan, SERPINF1, WNT5A, HGF, and FGF7 treatment compared to untreated control (UT). Scale bar = 100 μm. N=4 independent experiments.
b. Quantification of ISL1-EGFP+ cells (% of total DAPI+) after growth factor treatment for 3 days or in untreated control (UT). Boxes extend from 25th to 75th percentile, middle lines denote median, while whiskers show minimum and maximum values. N = 4 independent experiments. Statistical significance was evaluated with ANOVA one-way with Dunnett's multiple comparisons test and p-values for conditions significantly different from the UT control are shown.
c. Pancreatic M-E cell-derived growth factors induce C-peptide expression in EPs. Quantification of C-peptide+ out of total cells (DAPI+) after 3-day incubation of EPs with growth factors compared to untreated control (UT). Boxes extend from 25th to 75th percentile, middle lines denote median, while whiskers show minimum and maximum values. N = 4 independent experiments. Statistical significance was evaluated with ANOVA one-way with Dunnett's multiple comparisons test and p-values for conditions significantly different from the UT control are shown.
d. Quantification of INS+ cells induced from H1 hESC-derived EPs after growth factor treatment for 3 days. Boxes extend from 25th to 75th percentile, middle lines denote median, while whiskers show minimum and maximum values. N = 4 independent experiments. Statistical significance was evaluated with ANOVA one-way with Dunnett's multiple comparisons test and p-values for conditions significantly different from the untreated (UT) control are shown.
e. Representative flow cytometry plot with quantification of GCG+ cells in EPs cultured in media only (UT) or in the presence of Endocan.

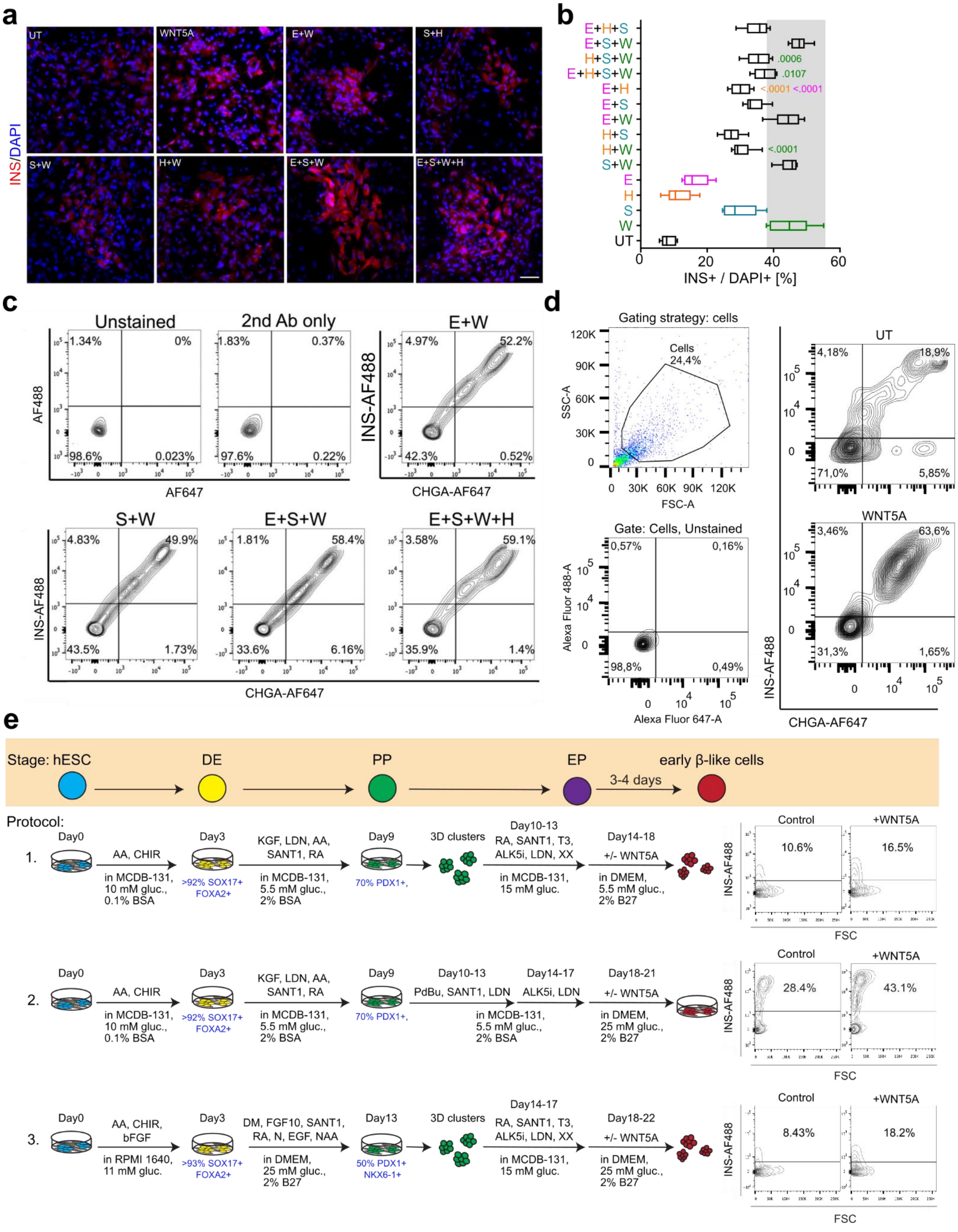

a
UT
WNT5A
E+W
S+H
S+W
H+W
E+S+W
E+S+W+H
INS/DAPI
b
E+H+S
E+S+W
H+S+W
E+H+S+W
E+H
E+S
E+W
H+S
H+W
S+W
E
H
S
W
UT
.0006
.0107
<.0001 <.0001
<.0001
INS+ / DAPI+ [%]
c
Unstained
2nd Ab only
E+W
S+W
E+S+W
E+S+W+H
AF488
AF647
INS-AF488
CHGA-AF647
d
Gating strategy: cells
Cells 24,4%
SSC-A
FSC-A
Gate: Cells, Unstained
Alexa Fluor 488-A
Alexa Fluor 647-A
UT
WNT5A
INS-AF488
CHGA-AF647
e
Stage: hESC
DE
PP
EP
3-4 days
early β-like cells
Protocol:
1.
Day0
AA, CHIR
in MCDB-131, 10 mM gluc., 0.1% BSA
Day3
>92% SOX17+ FOXA2+
KGF, LDN, AA, SANT1, RA
in MCDB-131, 5.5 mM gluc., 2% BSA
Day9
70% PDX1+,
3D clusters
Day10-13
RA, SANT1, T3, ALK5i, LDN, XX
in MCDB-131, 15 mM gluc.
Day14-18
+/- WNT5A
in DMEM, 5.5 mM gluc., 2% B27
Control
+WNT5A
10.6%
16.5%
INS-AF488
FSC
2.
Day0
AA, CHIR
in MCDB-131, 10 mM gluc., 0.1% BSA
Day3
>92% SOX17+ FOXA2+
KGF, LDN, AA, SANT1, RA
in MCDB-131, 5.5 mM gluc., 2% BSA
Day9
70% PDX1+,
Day10-13
PdBu, SANT1, LDN
Day14-17
ALK5i, LDN
in MCDB-131, 5.5 mM gluc., 2% BSA
Day18-21
+/- WNT5A
in DMEM, 25 mM gluc., 2% B27
Control
+WNT5A
28.4%
43.1%
INS-AF488
FSC
3.
Day0
AA, CHIR, bFGF
in RPMI 1640, 11 mM gluc.
Day3
>93% SOX17+ FOXA2+
DM, FGF10, SANT1, RA, N, EGF, NAA
in DMEM, 25 mM gluc., 2% B27
Day13
50% PDX1+ NKX6-1+
3D clusters
Day14-17
RA, SANT1, T3, ALK5i, LDN, XX
in MCDB-131, 15 mM gluc.
Day18-22
+/- WNT5A
in DMEM, 25 mM gluc., 2% B27
Control
+WNT5A
8.43%
18.2%
INS-AF488
FSC

Supplementary Figure 7. WNT5A promotes β-cell development in different differentiation protocols and in combination with other M-E cell-derived growth factors

a. INS (red) immunostainings after Endocan (E), SERPINF1 (S), WNT5A (W), and HGF (H) combinatorial treatment. Representative images for high concentration treatment are shown. Scale bar = 50 µm. N=6 independent experiments.

b. Quantification of INS+ cells of combinational treatment with Endocan (E, in red), SERPINF1 (S, in blue), HGF (H, in orange), and WNT5A (W, in green) compared to singular treatment and untreated control (UT). Boxes extend from 25th to 75th percentile, middle lines denote median, while whiskers show minimum and maximum values. N = 6 independent experiments. Statistical significance was evaluated with one-way ANOVA with Dunnett's multiple comparisons test and p-values for combinations significantly different from singular treatments are shown (colors indicate comparison group). The gray background highlights the highest % of INS+ cells indicating the most efficient treatments.

c. Representative flow cytometry plots of INS+ and CHGA+ cells after combinational growth factor 3-day long treatment of EPs. "Unstained" (no antibodies) and "2nd Ab only" (secondary antibodies only) samples were used to discriminate between positively stained and unstained populations in fluorescent channel plots. Similar gating strategy was used for other FC experiments described in the manuscript.

d. (Right panels) Representative flow cytometry plots of INS+ and CHGA+ cells after 3-day treatment of EPs with WNT5A or without treatment (UT). Left panels show gating strategy, i.e. cells were identified based on FSC-A vs SSC-A plot (left top panel) and the "Cells" gate was applied to fluorescent channels plots. "Unstained" (no antibodies, bottom left panel) or "2nd Ab only" (as in Suppl. Fig. c) were used to set up positively stained populations. Similar gating strategy was used throughout the manuscript.

e. Scheme of additional differentiation protocols (marked as 1-3) tested. Growth factors concentrations are listed in Table S3. Flow cytometry analysis was used to determine % of cells positive for marker proteins of consecutive differentiation stages, and these values are shown below the DE (for SOX17 and FOXA2) and PP stages (for NKX6.1 and PDX1), in blue. Representative flow plots of INS+ cells after 3-4 days of WNT5A treatment and untreated control are shown. AA = ActivinA, CHIR=CHIR99021, LDN=LDN193189, RA = Retinoic Acid, DM = Dorsomorphin, N = Noggin, NAA = Nicotinamide

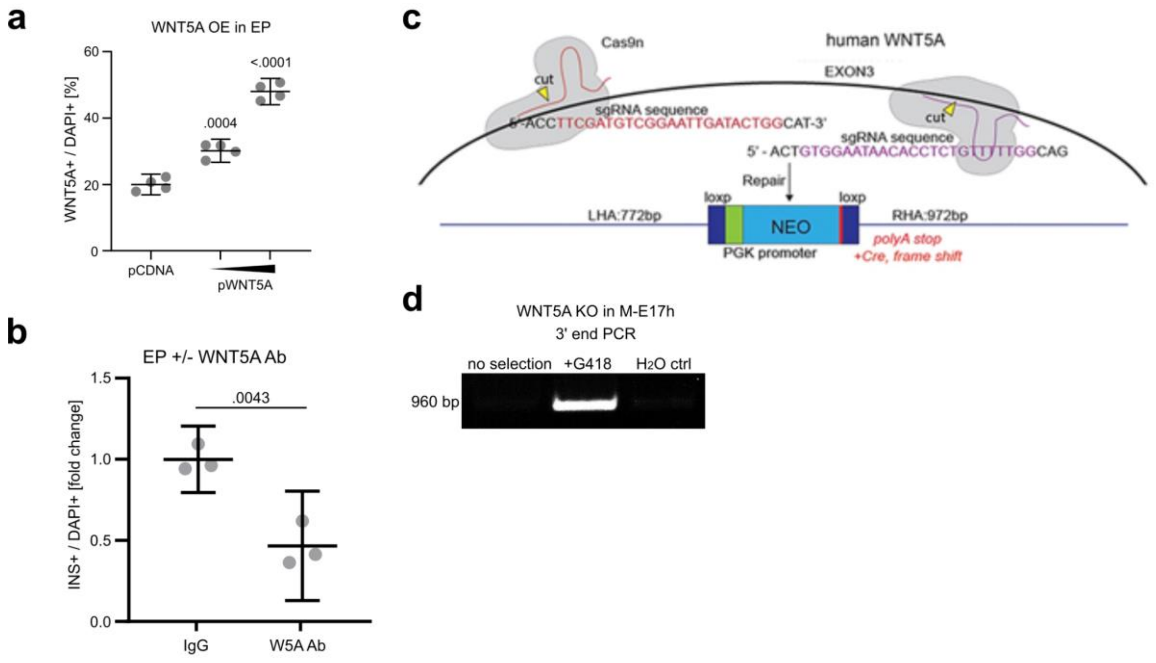

Supplementary Figure 8. WNT5A signaling in fetal human pancreas

a. Efficiency evaluation of WNT5A overexpression in hESC-derived EPs, corresponding to Figure 5C and D. Lines denote mean ± 95% CI and the statistical significance was estimated by unpaired two-tailed Student's t-test (p-values vs. pCDNA are shown). N = 4 independent experiments.
b. hESC-derived EPs treated with 1 μg of WNT5A neutralizing antibodies for 3 days. INS+ cells out of total (DAPI+) cells were quantified (N = 3 independent experiments) using immunofluorescence and the results are presented as fold change normalized to IgG treated control. Lines denote mean ± 95% CI and the statistical significance was estimated by unpaired two-tailed Student's t-test (p-value is shown).
c. Scheme of strategy to generate WNT5A knock-out in M-E17h and M-E20 cells using CRISPR-Cas9 nickase system.
d. PCR verification of WNT5A KO in M-E17h cells. PCR was performed using genomic DNA from control M-E17h cells and from targeted M-E17h cells after antibiotic (+G418) selection (WNT5A KO). The PCR primers bind to the 3'end-targeting site. Methods and primers were described previously (Yang et al., 2016). N=3 independent experiments.

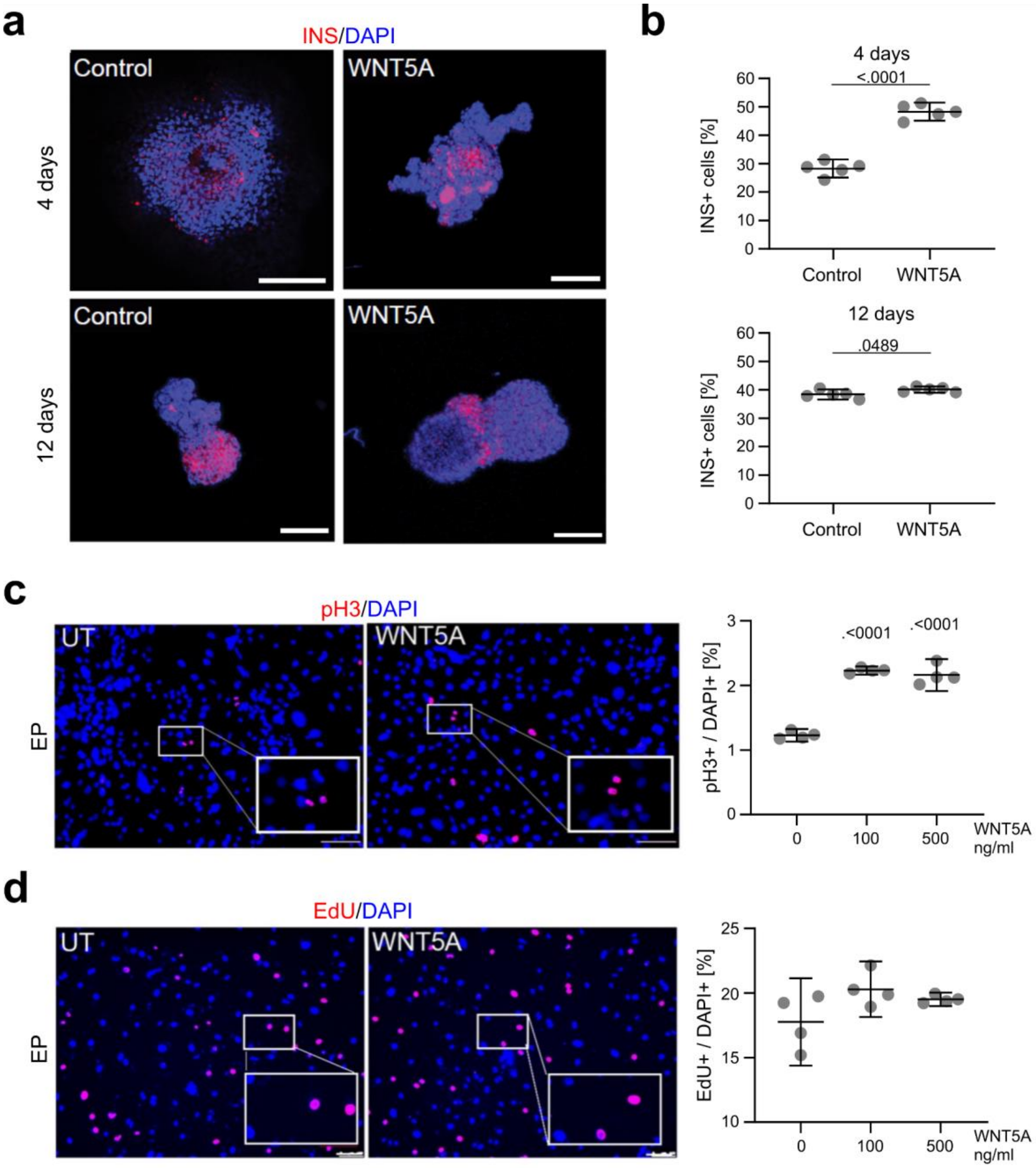
a
INS/DAPI
Control
WNT5A
4 days
12 days
b
4 days
<.0001
INS+ cells [%]
0
10
20
30
40
50
60
Control
WNT5A
12 days
.0489
c
pH3/DAPI
UT
WNT5A
EP
pH3+ / DAPI+ [%]
.<0001
0
100
500
WNT5A ng/ml
d
EdU/DAPI
UT
WNT5A
EP
EdU+ / DAPI+ [%]
10
15
20
25
0
100
500
WNT5A ng/ml

Supplementary Figure 9. WNT5A promotes EPs differentiation towards β-cells without proliferation induction

a. WNT5A facilitates INS+ (red) induction during 3D pancreatic differentiation. ISL1-EGFP hESCs were differentiated as 3D spheres (Pagliuca et al., 2014) and at the EP stage, cells were treated for two days either with T3, ALK5i or T3, ALK5i and WNT5A. Representative IF is shown at day 4 and 12 for control and WNT5A treated cells. Nuclei are shown in blue by DAPI. Scale bar = 100 µm. N = 5 independent experiments.
b. Quantification of INS+ cells out of total (DAPI+) cells after 4 and 12 days of the two-day WNT5A treatment of 3D EP clusters. While the proportion of INS+ cells is significantly higher in WNT5A-treated cells after 4 days, there is slight difference between the conditions after 12 days, as well as no increase in INS+ cells between 4 and 12 days in WNT5A conditions. Data is presented as mean ± 95% CI and p-values (unpaired two-tailed Student's t-test) are shown. N = 5 independent experiments.
c. WNT5A treatment has a modest effect on EP proliferation. EPs were stained with phospho-Histone H3 antibody (pH3, red) after 3 days of WNT5A treatment. Percentages of positive cells are shown in the right panel. Data is presented as mean ± 95% CI from 4 independent experiments. Statistical significance was evaluated with unpaired two-tailed Student's t-test. Scale bar =100 µm.
d. EdU cell proliferation assay after WNT5A treated EPs. EdU in green and DAPI in blue. Percentages of positive cells are shown in the right panel. Data is presented as mean ± 95% CI from 4 independent experiments. Statistical significance was evaluated with unpaired two-tailed Student's t-test. Scale bar = 100 µm.

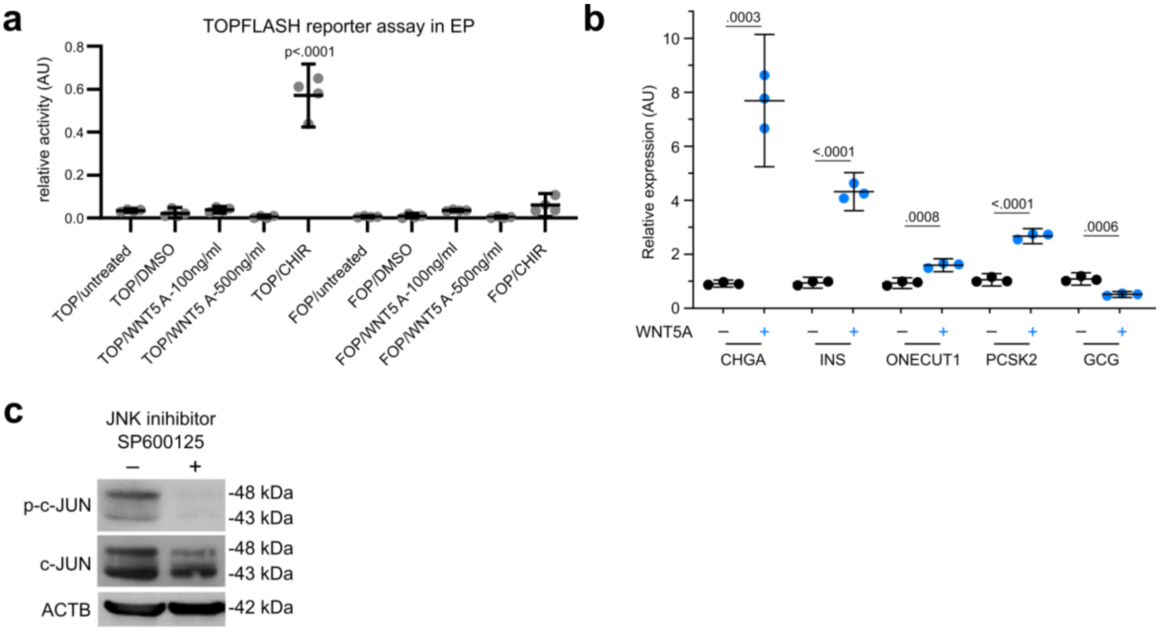

Supplementary Figure 10. WNT5A does not act through canonical WNT signaling while it activates JNK/JUN pathway in hESC-derived EPs

a. Activation of canonical WNT signaling was evaluated by TOPFLASH reporter assay. TOPFLASH (TOP) or FOPFLASH (FOP) plasmids were transfected to EPs for 48 hours and then cells were treated with DMSO (negative control), 100 or 500 ng/ml WNT5A, or positive control CHIR99021 (CHIR) for 3 days. pRLTK, which transcribed Renilla, was cotransfected together with TOPFLASH or FOPFLASH in all experimental groups as internal control. Ratios of Luciferase/Renilla are presented as mean ± 95% CI and significant p-values (multiple comparisons Tukey test vs. untreated - for WNT5A - or DMSO - for CHIR) are shown. N = 4 independent experiments.
b. qRT-PCR verification of selected RNA-sequencing results. Expression of *CHGA*, *INS*, *ONECUT1*, *PCSK2*, and *GCG*, was evaluated in 5 days untreated (-) and WNT5A-treated (+) EPs. The data is presented as mean ± 95% CI, N = 3, unpaired two-tailed t-test was used to determine p-values (shown).
c. Western blot analysis to confirm loss of c-JUN phosphorylation upon JNK inhibitor SP600125 treatment. Multiple bands of p-JNK and JNK correspond to 58 and 43 kDa isoforms. N= 4 independent experiments.

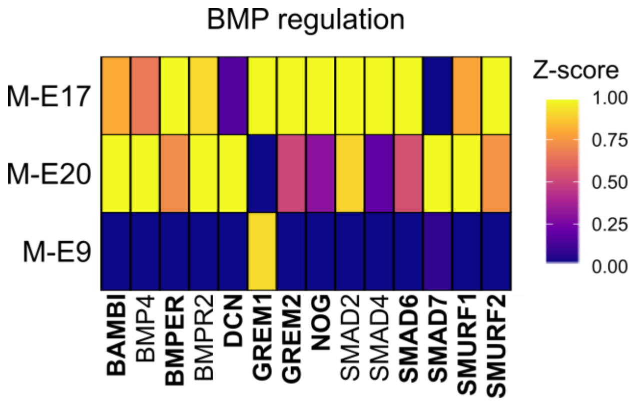

Supplementary Figure 11. M-E17 and M-E20 cells are enriched in BMP signaling antagonists

Heatmap presenting relative expression of selected genes involved in BMP regulation in M-E cells, based on RNA-Seq experiments presented in Fig. 3. BMP antagonist genes are highlighted in bold.

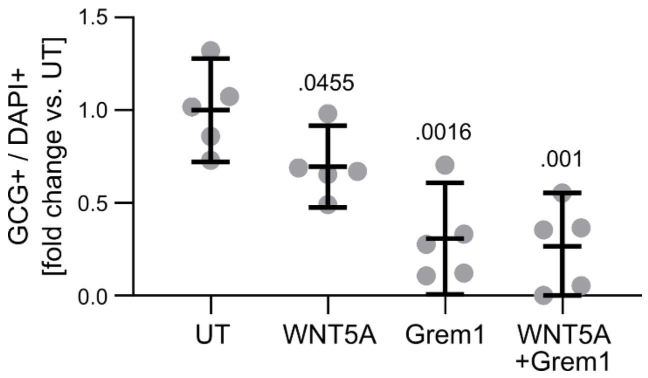

Supplementary Figure 12. WNT5A treatment combined with BMP inhibition reduces number of GCG+ cells

GCG+ cells were evaluated after three day EP treatment with WNT5A, Grem1, or in combination by immunofluorescence and the fold change in GCG+ cell number compared to untreated control (UT) is shown. The data is presented as mean ± 95% CI, n = 5 independent experiments. Statistical significance was determined using t-tests and p values are shown.

Supplementary Table 1. List of qRT-PCR primers

Gene	Primer sequence
VIM	tgcaggctcagattcaggaa<br>ctccggtactcagtggactc
PECAM1	tcccctaagaattgctgcca<br>ttcttcccaacacgccaatg
FSP1	aggggtgaagaagatgggtg<br>ccagtcacaccagcaatcac
FLK1	ttacttgcaggggacagagg<br>ttcccggtagaagcacttgt
CDH5	taccaggacgctttcaccat<br>aaaggctgctggaaaatggg
ICAM	agagaccccgttgcctaaaa<br>cagtacacggtgaggaaggt
VWF	tgcaacacttgtgtctgtcg<br>cgaaaggtcccagggttact
INS	agcctttgtgaaccaacacc<br>gctggtagagggagcagatg
PDX1	aagtctaccaaagctcacgcg<br>gtaggcgccgcctgc
WNT5A	ctccgctcggattcctc<br>caaagcaactcctgggctta
TBP	tgtgcacaggagccaagagt<br>atttcttgctgccagtctgg
GCG	aagcatttactttgtggctggatt<br>tgatctggatttctcctctgtgtct
BMP3	cagaaatacagtgtggcagaca<br>acacggttcgcagctttc
BMP4	ctcctagcaggacttggcat<br>tggctgtcaagaatcatgga
BMP6	tgcaggaagcatgagctg<br>gtgcgttgagtgggaagg
BMPER	ggacaggagagaatgggaca<br>tgtgtttgagggtgtgcagt
FZD3	tgccaactatgagagccatc<br>caacgtggatacaagaacgc
ONECUT1	tttttgggtgtgttgcctct<br>agaccttccggaggatgtg
PCSK2	tttcggtcaaatccttcctg<br>tgcaaaggccaagagaagac
SOX9	gtggtccttcttgtgctgc<br>gtacccgcacttgcacaac

Supplementary Table 2. List of primary antibodies

Antibody	Manufacturer	Catalog number	Dilution
Insulin (INS)	Dako	A0564	1:100
Glucagon (GCG)	Santa Cruz	Sc-7779	1:100
Chromogranin A (CHGA)	Abcam	Ab15160	1:100
C-peptide	DSHB	GN-ID4-s	1:100
GFP	Abcam	Ab13970	1:1000
PECAM1	DSHB	P2B1-c	1:100
VIMENTIN	Millipore	Ab5733	1:1000
WNT5A	Santa Cruz	Sc-23698	1:100
SOX17	R&D	AF1924	1:100(IF), 1:500 (FC)
FOXA2	Millipore	07-633	1:250
PDX1	R&D	AF2419	1:100
SOX9	MilliporeSigma	AB5535	1:100
NGN3	BCBC	RES4129	1:100
SST	Dako	A0566	1:400
NKX6.1	DSHB	F64A6B4	1:100
pH3	Millipore	06570	1:100
FZD3	Gift from Dr. Jeremy Nathans		1:100
p-JNK	Cell signaling	4668	1:1000
JNK	Cell signaling	9252	1:1000
p-c-JUN	Cell signaling	9261	1:1000
c-JUN	Cell signaling	9165	1:1000
p-Smad1/5	Cell signaling	9516	1:100
Beta-actin	Sigma-Aldrich	A5441	1:5000
Collagen IV	Milipore	AB756P	1:100
Laminin	Sigma-Aldrich	L9393	1:200
Alexa Fluor 488 Donkey Anti-Goat;	Jackson Immuno Research	705-545-147	1:400
Alexa Fluor 488 Donkey Anti-Rabbit	Jackson Immuno Research	711-545-152	1:400

Alexa Fluor 488 Donkey Anti-Chicken	Jackson Immuno Research	703-545-155	1:400
Alexa Fluor 488 Donkey Anti-Guinea Pig	Jackson Immuno Research	706-545-148	1:400
Alexa Fluor 488 Donkey Anti-Mouse	Jackson Immuno Research	715-545-150	1:400
Alexa Fluor 488 Donkey Anti-Sheep	Jackson Immuno Research	713-545-147	1:400
TRITC Donkey Anti-Goat	Jackson Immuno Research	705-025-147	1:400
TRITC Donkey Anti-Rabbit	Jackson Immuno Research	711-025-152	1:400
TRITC Donkey Anti-Mouse	Jackson Immuno Research	715-025-150	1:400
TRITC Donkey Anti-Guinea Pig	Jackson Immuno Research	706-025-148	1:400
TRITC Donkey Anti-Rat	Jackson Immuno Research	712-025-153	1:400
Alexa Fluor 647 Donkey Anti-Goat	Jackson Immuno Research	705-605-147	1:400
Alexa Fluor 647 Donkey Anti-Rabbit	Jackson Immuno Research	711-605-152	1:400
Sheep Anti-Mouse IgG - Horseradish Peroxidase	GE Healthcare	RPN4201	1:1000
Goat Anti-Rabbit IgG - Horseradish Peroxidase	GE Healthcare	RPN4301	1:1000

Supplementary Table 3. Growth factors and small molecules used in Figures 4-7 and Figures S2-3 and S6-12.

Growth factors	Manufacturer	Concentration 1	Concentration 2
Y-27632	Stemgent	10 µM	
bFGF	R&D	4 ng/ml (hESC media)	5 ng/ml (differentiation protocol 3 in Figure S7e)
ActivinA (AA)	R&D	100 ng/ml (differentiation Days 1-3)	20 ng/ml (differentiation Days 4-9)
CHIR99021 (CHIR)	Stemgent	3 µM	
Ascorbic acid (VitC)	Sigma-Aldrich	44 mg/l	50 µg/ml (differentiation protocol 3 in Figure S7e)
KGF	Peprotech	12.5 ng/ml	
Dorsomorphin (DM)	Stemcell Technologies	0.75 µM	
FGF10	R&D	50 ng/ml	
EGF	R&D	100 ng/ml	
Retinoic Acid (RA)	Sigma-Aldrich	2 µM	
SANT-1	Sigma-Aldrich	0.25µM	
Noggin (N)	R&D	100 ng/ml	
LDN193189 (LDN)	Stemgent	200 nM	
Nicotinamide (NAA)	Sigma-Aldrich	10 mM	
PdBU	Sigma-Aldrich	1 µM	
AlK5i	Axxora	1 µM	
XX	EMD Millipore	1 mM	
T3	Sigma-Aldrich	1 mM	
FGF7	Peprotech	50 ng/ml	100 ng/ml
HGF	R&D	50 ng/ml	100 ng/ml
PDPN	R&D	100 ng/ml	500 ng/ml
SERPINF1	R&D	500 ng/ml	1 µg/ml
WNT5A	R&D	100 ng/ml	500 ng/ml
LIF	Home-made	~0.5 U/ml	~1 U/ml
EGF	R&D	25 ng/ml	50 ng/ml
THBS2	R&D	1 µg/ml	5 µg/ml
IGF1	R&D	10 ng/ml	50 ng/ml
Endocan	R&D	10 ng/ml	50 ng/ml
WNT3A	R&D	20 ng/ml	40 ng/ml
Gremlin1	R&D	50 ng/ml	200 ng/ml
SP600125	EMD Millipore	20 µM	40 µM
BMP4	R&D	200 ng/ml	
Anisomycin	Sigma	1 ng/µl	

Supplementary Table 4. Growth factor combinations used in Figure S7.

Growth Factors	Concentration 1	Concentration 2
E+S	1 ng/ml Endocan + 500 ng/ml SERPINF1	1 ng/ml Endocan + 1 μg/ml SERPINF1
E+W	1 ng/ml Endocan + 100 ng/ml WNT5A	1 ng/ml Endocan + 500 ng/ml WNT5A
E+H	1 ng/ml Endocan + 50 ng/ml HGF	1 ng/ml Endocan + 100 ng/ml HGF
S+W	1 μg/ml SERPINF1 + 100 ng/ml WNT5A	1 μg/ml SERPINF1 + 500 ng/ml WNT5A
S+H	1 μg/ml SERPINF1 + 50 ng/ml HGF	1 μg/ml SERPINF1 + 100 ng/ml HGF
W+H	100 ng/ml WNT5A + 50 ng/ml HGF	500 ng/ml WNT5A + 50 ng/ml HGF
E+H+S	1 ng/ml Endocan + 50 ng/ml HGF + 1 μg/ml SERPINF1	1 ng/ml Endocan + 100 ng/ml HGF + 1 μg/ml SERPINF1 + 500 ng/ml WNT5A
E+S+W	1 ng/ml Endocan + 1 μg/ml SERPINF1 + 100 ng/ml WNT5A	1 ng/ml Endocan + 1 μg/ml SERPINF1 + 500 ng/ml WNT5A
H+S+W	50 ng/ml HGF + 1 μg/ml SERPINF1 + 100 ng/ml WNT5A	100 ng/ml HGF + 1 μg/ml SERPINF1 + 100 ng/ml WNT5A
E+H+S+W	1 ng/ml Endocan + 50 ng/ml HGF + 1 μg/ml SERPINF1 + 100 ng/ml WNT5A	1 ng/ml Endocan + 100 ng/ml HGF + 1 μg/ml SERPINF1 + 500 ng/ml WNT5A

Supplementary Table 5. Characteristics of human islet donors.

Number	Age	Sex	BMI	Cause of death
1	54	female	35.9	CVA/Stroke
2	67	female	30.8	CVA/Stroke
3	41	male	30.5	Anoxia
4	49	male	28.5	heart attack
5	63	male	30.6	CVA/Stroke
6	69	male	31.3	CVA/Stroke
