# Supplementary material for: Human pancreatic microenvironment promotes β-cell differentiation via non-canonical WNT5A/JNK and BMP signaling
Source: Nat Commun. 2022 Apr 12;13:1952. doi: 10.1038/s41467-022-29646-1 (PMC9005503; doi:10.1038/s41467-022-29646-1)
Supplement: Supplementary file 3 — Reporting Summary [file 41467_2022_29646_MOESM3_ESM.pdf]

Corresponding author(s): Malgorzata Borowiak

Last updated by author(s): Mar 7, 2022

Reporting Summary

Nature Portfolio wishes to improve the reproducibility of the work that we publish. This form provides structure for consistency and transparency in reporting. For further information on Nature Portfolio policies, see our Editorial Policies and the Editorial Policy Checklist.

Statistics

For all statistical analyses, confirm that the following items are present in the figure legend, table legend, main text, or Methods section.

n/a	Confirmed	
☐	☒	The exact sample size (*n*) for each experimental group/condition, given as a discrete number and unit of measurement
☐	☒	A statement on whether measurements were taken from distinct samples or whether the same sample was measured repeatedly
☐	☒	The statistical test(s) used AND whether they are one- or two-sided<br>*Only common tests should be described solely by name; describe more complex techniques in the Methods section.*
☐	☒	A description of all covariates tested
☐	☒	A description of any assumptions or corrections, such as tests of normality and adjustment for multiple comparisons
☐	☒	A full description of the statistical parameters including central tendency (e.g. means) or other basic estimates (e.g. regression coefficient) AND variation (e.g. standard deviation) or associated estimates of uncertainty (e.g. confidence intervals)
☐	☒	For null hypothesis testing, the test statistic (e.g. *F*, *t*, *r*) with confidence intervals, effect sizes, degrees of freedom and *P* value noted<br>*Give P values as exact values whenever suitable.*
☒	☐	For Bayesian analysis, information on the choice of priors and Markov chain Monte Carlo settings
☒	☐	For hierarchical and complex designs, identification of the appropriate level for tests and full reporting of outcomes
☒	☐	Estimates of effect sizes (e.g. Cohen's *d*, Pearson's *r*), indicating how they were calculated

Our web collection on statistics for biologists contains articles on many of the points above.

Software and code

Policy information about availability of computer code

Data collection	LAS X software v5.0 (Leica), FACSDiva v8.0 (BD), Amnis ImageStreamX MarkII (Luminex); CFX manager software v3.1 (Applied Biosystems); CLARIOstar illuminometer
Data analysis	LAS X software v5.0 (Leica), FlowJo v10.4.2; ImageJ v1.5 (NIH), GraphPad Prism v8.4; CFX manager software v3.1 (Applied Biosystems); IDEAS software v6.0 (Millipore); TopHat v2.1.1 (https://ccb.jhu.edu/software/tophat/index.shtml), Cufflinks v2.2.1 (https://github.com/cole-trapnell-lab/cufflinks), HTseq v 1.0.0 (http://www-huber.embl.de/users/anders/HTSeq), GSEA v4.0 (Broad Institute, http://software.broadinstitute.org/gsea/index.jsp), TFactS v2 (http://www.tfacts.org/TFactS-new/TFactS-v2/index1.html), BioVenn (http://www.biovenn.nl/)

For manuscripts utilizing custom algorithms or software that are central to the research but not yet described in published literature, software must be made available to editors and reviewers. We strongly encourage code deposition in a community repository (e.g. GitHub). See the Nature Portfolio guidelines for submitting code & software for further information.

Data

Policy information about availability of data

All manuscripts must include a data availability statement. This statement should provide the following information, where applicable:

- Accession codes, unique identifiers, or web links for publicly available datasets
- A description of any restrictions on data availability
- For clinical datasets or third party data, please ensure that the statement adheres to our policy

The authors declare that all data supporting the findings of this study are available within the article, supplementary information files and the Source Data. Raw RNA-Seq data generated during the study have been deposited in the GEO Repository under accession number GSE90785. Raw image files are available from

the corresponding author upon a reasonable request.

Field-specific reporting

Please select the one below that is the best fit for your research. If you are not sure, read the appropriate sections before making your selection.

- [x] Life sciences
- [ ] Behavioural & social sciences
- [ ] Ecological, evolutionary & environmental sciences

For a reference copy of the document with all sections, see nature.com/documents/nr-reporting-summary-flat.pdf

Life sciences study design

All studies must disclose on these points even when the disclosure is negative.

Sample size	Sample size was determined based on Authors' experience and preliminary experiments as well as similar experiments in the published literature. Sample size caluclations were performed in G*Power. The conclusions of our research were made based on multiple complementary approaches.
Data exclusions	No data was excluded from the analyses.
Replication	In all cell culture experiments at least 3 independent experiments were performed. For immunofluorescence experiments quantitative analysis at least five randomly selected images were counted per replicate. The reproducibility was also assesed by use of various hPSC lines (3 lines), various differentiation protocols (5 protocols), various molecular biology methods to verify hypotheses, including rescue experiments, e.g. WNT5A induction of INS+ cells was assesed using recombinant protein, M-E knock-outs, overexpression, and antibody blocking. Results were replicated by independent researchers.
Randomization	Randomization was not performed as it is not applicable in in vitro differentiation experiments, where all samples within the biological experiment are derived from the same batch of cells.
Blinding	Investigators were blinded to sample identity during data collection and analysis, where these could induce a bias (e.g. all experiments with quantifcation of microscopic images). In experiments with factors screeening, factors were coded during experiments and data collection, and images were quantified by independent, blinded investigators. Data were acquired using imaging setups that perforemd the measurement independently of the observer.

Reporting for specific materials, systems and methods

We require information from authors about some types of materials, experimental systems and methods used in many studies. Here, indicate whether each material, system or method listed is relevant to your study. If you are not sure if a list item applies to your research, read the appropriate section before selecting a response.

Materials & experimental systems

n/a	Involved in the study	
	x	Antibodies
	x	Eukaryotic cell lines
x		Palaeontology and archaeology
	x	Animals and other organisms
	x	Human research participants
x		Clinical data
x		Dual use research of concern

Methods

n/a	Involved in the study	
x		ChIP-seq
	x	Flow cytometry
x		MRI-based neuroimaging

Antibodies

Antibodies used

Insulin;Dako;A0564;1:100
Glucagon;Santa Cruz;Sc-7779;1:100
Chromogranin A;Abcam;Ab15160;1:100
C-peptide;DSHB;GN-ID4-s;1:100
GFP;Abcam;Ab13970;1:1000
PECAM1;DSHB;P2B1-c;1:100
VIMENTIN;Millipore;Ab5733;1:1000
WNT5A;Santa Cruz;Sc-23698;1:100
SOX17; R&D; AF1924; 1:100(IF), 1:500 (FC)
FOXA2; Millipore; 07-633; 1:250
PDX1;R&D;AF2419;1:100
SOX9;MilliporeSigma;AB5535;1:100
NGN3; BCBC; RES4129; 1:100

SST;Dako;A0566;1:400
NKX6.1;DSHB;F64A6B4;1:100
pH3;Millipore ;6570;1:100
FZD3;Gift from Dr. Jeremy Nathans;1:100
p-JNK;Cell signaling;4668;1:1000
JNK;Cell signaling;9252;1:1000
p-c-JUN;Cell signaling;9261;1:1000
p-Smad1/5;Cell signaling;9516;1:100
Beta-actin;Sigma-Aldrich;A5441;1:5000
Collagen IV;Milipore;AB756P;1:100
Laminin;Sigma-Aldrich;L9393;1:200
Alexa Fluor 488 Donkey Anti-Goat; Jackson Immuno Research (JIR); 705-545-147
Alexa Fluor 488 Donkey Anti-Rabbit; JIR; 711-545-152
Alexa Fluor 488 Donkey Anti-Chicken; JIR; 703-545-155
Alexa Fluor 488 Donkey Anti-Guinea Pig; JIR; 706-545-148
Alexa Fluor 488 Donkey Anti-Mouse; JIR; 715-545-150
Alexa Fluor 488 Donkey Anti-Sheep; JIR; 713-545-147
TRITC Donkey Anti-Goat; JIR; 705-025-147
TRITC Donkey Anti-Rabbit; JIR; 711-025-152
TRITC Donkey Anti-Mouse; JIR; 715-025-150
TRITC Donkey Anti-Guinea Pig; JIR; 706-025-148
TRITC Donkey Anti-Rat; JIR; 712-025-153
Alexa Fluor 647 Donkey Anti-Goat; JIR; 705-605-147
Alexa Fluor 647 Donkey Anti-Rabbit; JIR; 711-605-152

Validation

Antibodies profiles are available at Resource Identification Portal (RRID, https://scicrunch.org/resources). All antibodies have been obtained from respected manufacturers that provide detailed validation, and were validated for use in human cells.
Anti-Insulin;Dako;A0564;1:100; RRID: AB_10013624; https://www.citeab.com/antibodies/3382917-a0564-insulin; validated for use in human pluripotent stem cell-derived β-cells in IF in Yang et al., Cell Stem Cell 2020 (doi: 10.1016/j.stem.2020.06.015)
Glucagon antibody (C-18); Santa Cruz;Sc-7779;1:100; RRID: AB_641024;
Anti-Chromogranin A;Abcam;Ab15160;1:100; RRID: AB_301704;
https://www.abcam.com/chromogranin-a-antibody-ab15160.html
Insulin (pro-)/C-peptide antibod;DSHB;GN-ID4-s;1:100; RRID: AB_2255626;
https://dshb.biology.uiowa.edu/GN-ID4; validated e.g., by Asadi 2015 (doi: 10.1369/0022155415576541) who performed IF in human tissue as co-staining with multiple other anti-insulin antibodies.
Anti-GFP;Abcam;Ab13970;1:1000; RRID: AB_300798;
https://www.abcam.com/gfp-antibody-ab13970.html; validated for IF by supplier using GFP-transfected NIH/3T3 cells
Anti-PECAM1 (CD31);DSHB;P2B1-c;1:100; RRID: AB_528430; https://dshb.biology.uiowa.edu/P2B1: "Recommended Applications: Function Blocking, Immunofluorescence, Immunohistochemistry", "Immunoprecipitation; Positive Tested Species Reactivity: Human"
Anti-Vimentin;Millipore;Ab5733;1:1000; RRID: AB_11212377;
https://www.merckmillipore.com/PL/pl/product/Anti-Vimentin-Antibody,MM_NF-AB5733
Wnt-5a Antibody (C-16);Santa Cruz;Sc-23698;1:100; RRID: AB_2215601; We have validated this antibody for IF in human cells by overexpression (Fig. 5c) and knock-out (Fig. 5e) in the current manuscript.
Human SOX17 Antibody; R&D; AF1924; 1:100(IF), 1:500 (FC); RRID: AB_355060;
https://www.rndsystems.com/products/human-sox17-antibody_af1924: supplier's page includes over 200 references for the antibody use, including IF and FC in human cells. Validated by differentiation of human pluripotent cells (SOX17-) into definitive endoderm (SOX17+).
Anti-HNF3β/FOXA2 Antibody; Millipore; 07-633; 1:250; RRID: AB_390153; IF-validated in human cells using CRISPRi by Genga et al., Cell Reports 2019, doi: 10.1016/j.celrep.2019.03.076
Human PDX-1/IPF1 Antibody; R&D; AF2419; 1:100; RRID: AB_355257;
https://www.rndsystems.com/products/human-pdx-1-ipf1-antibody_af2419: "PDX-1/IPF1 was detected in immersion fixed BG01V human embryonic stem cells differentiated into pancreatic progenitor cells (...) Specific staining was localized to nuclei."
Anti- SOX9; Millipore Sigma; AB5535; 1:100; RRID: AB_2239761;
https://www.merckmillipore.com/PL/pl/product/Anti-Sox9-Antibody,MM_NF-AB5535#: "species reactivity: human", "Anti-Sox9 Antibody is a well characterized affinity purified Rabbit Polyclonal Antibody that reliably detects Transcription Factor Sox-9."
Polyclonal Human Ngn3 raised in Sheep - Antibody RES4129; Beta Cell Biology Consortium; RES4129; 1:100; RRID: AB_2149527;
http://www.betacell.org/pdf/betacell-resource-4129.pdf; validated for FC in human endocrine progenitors by Sharon et al., Cell Reports 2019 (doi: 10.1016/j.celrep.2019.04.083)
Polyclonal Rabbit Anti-Human Somatostatin antibody; Dako; A0566; 1:400; RRID: AB_2688022; validated e.g. in Yang et al., Cell Stem Cell 2020 (doi: 10.1016/j.stem.2020.06.015)
Nkx6.1 Antibody; DSHB;F64A6B4; 1:100; RRID: AB_532380; https://dshb.biology.uiowa.edu/F64A6B4: "Positive Tested Species Reactivity: Human", "Recommended Applications: FACS, Immunohistochemistry","IHC note: early embryonic pancreatic epithelium; ventral neural tube; stomach mesenchyme and adult β cells."
Anti-phospho-Histone H3 (Ser10) Antibody, Mitosis Marker ;Millipore ;6570;1:100; RRID: AB_310177; https://www.merckmillipore.com/PL/pl/product/Anti-phospho-Histone-H3-Ser10-Antibody-Mitosis-Marker,MM_NF-06-570; IF-validated in human cells by e.g., Stojic et al., Nat. Comm. 2020 (doi: 10.1038/s41467-020-14978-7)
FZD3; created in Dr. Jeremy Nathans lab; 1:100; validated with KO by Jeremy Nathan's lab (e.g. in 10.1523/JNEUROSCI.4698-05.2005)
Phospho-SAPK/JNK (Thr183/Tyr185) (81E11) Rabbit mAb antibody; Cell Signaling; 4668; 1:1000; RRID: AB_823588; https://www.cellsignal.com/products/primary-antibodies/phospho-sapk-jnk-thr183-tyr185-81e11-rabbit-mab/4668; Validated in human cells in WB.

SAPK/JNK Antibody; Cell Signaling; 9252; 1:1000; RRID: AB_2250373; https://www.cellsignal.com/products/primary-antibodies/sapk-jnk-antibody/9252: Validated in human cells in WB
Phospho-c-Jun (Ser63) II Antibody; Cell Signaling; 9261; 1:1000; RRID: AB_2130162; https://www.cellsignal.com/products/primary-antibodies/phospho-c-jun-ser63-ii-antibody/9261; validated for WB in human cells; validated for IF in human cells in Fallachi-Sichani et al., Mol Syst Biol 2015 (10.15252/msb.20145877) by small molecule modulation of JNK pathway.
Phospho-Smad1/5 (Ser463/465) (41D10) Rabbit mAb; Cell Signaling; 9516; 1:100; RRID: AB_491015; https://www.cellsignal.com/products/primary-antibodies/phospho-smad1-5-ser463-465-41d10-rabbit-mab/9516: Validated for IF in human cells
Anti-β-Actin; Sigma-Aldrich; A5441; 1:5000; RRID: AB_476744; https://www.sigmaaldrich.com/PL/pl/product/sigma/a5441: validated for human cells in western blot; validated by knockdown in Chang et al., Scientific Reports 2017 (doi: 10.1038/s41598-017-16390-6)
Anti-Collagen Antibody, Type IV; Milipore; AB756P;1 :100; RRID: AB_2276457; https://www.merckmillipore.com/PL/pl/product/Anti-Collagen-Antibody-Type-IV,MM_NF-AB756P; validated for IF in human cells by Rybinski et al., Oncotarget 2015 (doi: 10.18632/oncotarget.4559)
Anti-Laminin antibody produced in rabbit; Sigma-Aldrich; L9393; 1:200; RRID: AB_477163; https://www.sigmaaldrich.com/PL/pl/product/sigma/l9393: "By indirect immunofluorescence the antibody demonstrates specific basement membrane staining of enzymatically unmasked human and animal tissue."

Eukaryotic cell lines

Policy information about cell lines

Cell line source(s)	ISL1-EGFP (a gift from Dr. Kenneth Chien and Dr. Lei Bu),<br>Hues8 (Harvard Stem Cell Institute, RRID:CVCL_B207),<br>H1 (Wicell, WA-01, RRID:CVCL_9771),<br>HUVECs (ATCC),<br>MS1 (ATCC)<br>MEF (derived from e12.5 ICR/CD1 embryos from Taconic)
Authentication	Cell lines were obtained directly from the vendors. Cell lines were authenticated based on qPCR and IF analysis of marker genes or expression, and in case of ISL1-EGFP by reporter expression.
Mycoplasma contamination	All cells used in the study were negative for Mycoplasma, as assessed be routinely performed RT-PCR analysis of culture media
Commonly misidentified lines (See ICLAC register)	None

Animals and other organisms

Policy information about studies involving animals; ARRIVE guidelines recommended for reporting animal research

Laboratory animals	ICR/CD1 6-12 week females purchased from Taconic for MEF derivation from e12.5 embryos
Wild animals	N/A
Field-collected samples	N/A
Ethics oversight	Animal studies were regulated and approved by the IACUC at Baylor College of Medicine, protocol AN-6325 to MB.

Note that full information on the approval of the study protocol must also be provided in the manuscript.

Human research participants

Policy information about studies involving human research participants

Population characteristics	Human islets were obtained from heart-beating, brain-dead or deceased, non-diabetic human donors from Isolation Center at Methodist Research Institute, Houston, Texas. All samples were deidentified and we did not obtain any personal information.
Recruitment	No participants were recruited. Tissue was harvested from brain-dead or deceased donors.
Ethics oversight	Human islets were obtained with informed consent for transplant or research use from relatives of donors, under Human Islet Isolation for Research (HIIR) protocol: Pro00001097 to O.S at Methodist Research Institute. The study design and conduct complied with all relevant regulations regarding the use of human study participants and was conducted in accordance with the criteria set by the Declaration of Helsinki.

Note that full information on the approval of the study protocol must also be provided in the manuscript.

Flow Cytometry

Plots

Confirm that:

☒ The axis labels state the marker and fluorochrome used (e.g. CD4-FITC).

☒ The axis scales are clearly visible. Include numbers along axes only for bottom left plot of group (a 'group' is an analysis of identical markers).

☒ All plots are contour plots with outliers or pseudocolor plots.

☒ A numerical value for number of cells or percentage (with statistics) is provided.

Methodology

Sample preparation	Cells were dissociated, washed with PBS, filtered through a 40 µm cell strainer and fixed with 4% PFA for 30 min at 4°C. 1% BSA, 0.1% saponin in PBS was used to dilute antibodies and to permeabilize cells. Primary antibodies are described in Table S2 for overnight incubation at 4°C rotor. After primary antibody incubation, samples were washed with 1% BSA, 0.1% saponin in PBS once and spun down at 1,200 g for 5 min. Secondary fluorophore conjugated antibodies were incubated overnight at 4°C. Then cells were centrifuged at 1,200 g for 5 min and washed 1% BSA, 0.1% saponin in PBS and filtered through a 40 µm cell strainer before flow cytometry.
Instrument	LSRII (BD Biosciences)
Software	BD FACSDiva (BD Biosciences), FlowJo
Cell population abundance	Minimum 5000 singlet cells were captured for each sample analysed.
Gating strategy	Cells were gated based on FSC-A vs. SSC-A plots for debris exclusion, then singlets were gated using FSC-W vs. FSC-H plots to discriminate doublets. Positive IF staining was gated based on II Ab and no-Ab controls, as well as cells from earlier differentiation stage that were negative for analyzed markers.

☒ Tick this box to confirm that a figure exemplifying the gating strategy is provided in the Supplementary Information.
